# Two-point discrimination values vary depending on test site, sex and test modality in the orofacial region: a preliminary study

**DOI:** 10.1590/1678-7757-2016-0462

**Published:** 2017

**Authors:** Sang-Yeun WON, Hye-Kyoung KIM, Mee-Eun KIM, Ki-Suk KIM

**Affiliations:** 1 Department of Oral Medicine, Dankook University College of Dentistry, Cheonan, South Korea

**Keywords:** Facial, Oral, Location, Sex, Modality, Perception

## Abstract

The two-point discrimination (TPD) test is one of the most commonly used neurosensory tests to assess mechanoperception in the clinical settings. While there have been numerous studies of functional sensibility of the hand using TPD test, there have been relatively not enough reports on TPD in the orofacial region.

**Objective:**

The aims of the present study were to determine the normal values of TPD in the six trigeminal sites (the forehead, cheek, mentum, upper lip, lower lip, and the tongue tip) and to investigate the effect of the site, sex, and test modality on the TPD perception.

**Material and Methods:**

Forty healthy volunteers consisting of age-matched men (20) and women (20) with a mean age of 27.1 years were recruited. One examiner performed the TPD test using a simple hand-operated device, i.e., by drawing compass with a blunt or sharp-pointed tip. The static TPD with a blunt-pointed tip (STPDB), moving TPD with a blunt-pointed tip (MTPDB), and static TPD with a sharp-pointed tip (STPDS) were measured. The predictors were the site, sex, and test modality, and the outcome variable was the TPD value. Three-way ANOVA was used for statistics.

**Results:**

The analysis showed a significant effect of the site, sex and test modality on the TPD values. Significant differences between the test sites were observed with the descending order from the forehead and cheek>mentum>upper lip and lower lip>tongue tip and index finger. Women showed lower TPD values than those of men. The STPDS measurements were consistently lower than those of the STPDB and MTPDB.

**Conclusions:**

The normal values of TPD in this study suggest that the cheek and forehead were less sensitive than other regions evaluated and women were more sensitive than men. The STPDS was the most sensitive test modality.

## Introduction

Clinical neurosensory testing is performed to evaluate sensory abnormalities. Routinely conducted tests for the assessment of altered sensation include three levels of tests, i.e., spatiotemporal perception, contact detection and nociception or temperature^30^. Above all, the most critical sensory test is related to touch perception rather than nociception, i.e., a large myelinated A-fiber function^5^.

The primary stimuli for tactile sensation are touch, pressure and vibration applied to skin, and mechanoreceptors are sensitive to the skin deformation caused by mechanical pressure^28^. Various traditional techniques such as the Semmes-Weinstein nylon monofilaments for pressure perception, tuning forks for the vibration thresholds and two-point discrimination (TPD) tests have been used for measuring the sensitivity of mechanoreceptors^5,6,26^. Above all, TPD is widely used by clinicians due to its simplicity^9^. Weber first introduced TPD in 1853 and defined it as “the distance between compass points necessary to feel two contacts”^13^. Dellon, Mackinnon and Crosby^7^ (1987) have reported the TPD tests have interobserver reliability. While Jerosch-Herold^9^ (2000) thought that TPD lacks sensitivity. Such controversy comes from a lack of standardized protocol for determining end-point distance of TPD^13^. Moberg^14^ (1990) also stated that valid and repeatable results of TPD test depend on exact protocol and proper tools. Despite controversy regarding the test reliability, TPD is one of the most commonly used clinical tests due to its simplicity for evaluation of peripheral nerve injury and sensory recovery after nerve damage or repair^3,9,10,13^. Furthermore, there are not enough tools for clinicians to assess tactile acuity in the clinical setting. In this respect, TPD test is still a valuable technique and should not be underestimated as a exploration tool for functional sensation.

There are various factors that can influence two-point discrimination values including test site, sex, test modality, age, device, and applied force^3,10,25,26^. It is well established that spatial acuity varies from one body site to another^24^. Notably, oral region, such as the lip and tongue and finger have superior spatial acuity, i.e., the sensory neural pathways innervating these regions are specialized for spatial information processing^24^. Therefore, it is no wonder that damage on these sensory nerves is likely to bring a prominent loss of sensory acuity^24^. Accordingly, accurate measurement of orofacial spatial resolution deserves the attention of clinicians.

The modality of touch in TPD could be classified into three: static two-point discrimination with blunt tip, moving two-point discrimination with blunt tip, and static two-point discrimination with sharp tip^12,13,26^. Static and moving TPD with blunt tip is usually tested using the Disk-Criminator and the Aesthesiometer is used for static TPD with sharp tip^7,26^.

While there have been numerous studies of functional sensibility of the hand using TPD test, there have been relatively not enough reports on TPD in the orofacial region. In addition, it is not easy to use the various test tools such as Disk-criminator and Aesthesiometer for different test modalities in the clinical settings. Thus, the purpose of this study was to determine the normal values of two-point discrimination using a simple hand-operated device in the orofacial region and compare the sensitivities of two-point discrimination by the test site, sex, and test modality. The mandibular nerve-innervated area, compared to other regions, was hypothesized to show spatial acuity in the TPD perception. Additionally, we hypothesized that women are more sensitive to TPD than men and the TPD test with moving or sharp tip affects the TPD perception.

## Material and methods

### Participants

An advertisement on the experiment was posted in the dental hospital and dental school. The exclusion criteria of this study excluded those who had neurologic disorders, chronic pain, sleep disorders, and systemic diseases such as uncontrolled hypertension or diabetes. Of 52 volunteers from the dental school, we excluded volunteers who had temporomandibular disorder, sleep disorder and numbness after orthognathic surgery. A total of 42 subjects (20 men, 22 women) remained after the exclusion process. To match sex ratio, 20 subjects among 22 women were randomly selected. A total of 40 healthy volunteers from the dental school of Dankook University (20 women, 20 men) aged 21 to 37 years (mean age of 27.1 years, S.D. 3.0) participated in the study. This study was performed in accordance with the Helsinki Declaration and the University Institutional Review Board approved the study (IRB No H-1303/004/003). Written informed consent was obtained from all subjects after full explanation of the objectives and procedures of the study.

As a pilot test, we used Lehr’s formula^11^ to calculate the sample size for a power of 80% and a two-sided significance level of 0.05. We performed a pilot test on the cheek and forehead of five participants. Then, we estimated the difference in means and standard deviation of the two sites and calculated the standardized difference. Assuming that the difference in means is 1.7 and the standard deviation is 1.9, we would require approximately 20 patients.

### Test sites and modality

The test sites were defined as the three major sensory branches of the trigeminal nerve region corresponding to the ophthalmic branch (V1), the maxillary branch (V2) and the mandibular branch (V3). In these three branches, six coordinates were selected for the experiment. These were the mentum (above the mental foramen); the vermilion of the lower lip; the vermilion of the upper lip; the tip of the tongue; the mid-point of the cheek and the forehead (2 cm above the midpoint of the brow). The index fingertip was chosen randomly between the left and right side and was tested to examine the sensory sensitivity of the subjects and for comparison with the orofacial region. The testing was performed starting with the index finger, then proceeding to the six orofacial test sites in random order, selecting alternatively from the right and left side. To select the test site randomly, the examiner put the papers on which test sites were written in a box and picked a paper before the test. The test sites were chosen according to the site written on the selected paper.

Three modalities of TPD tests were performed bilaterally at randomly selected trigeminal test sites. The static two-point discrimination with blunt-pointed tip (STPDB), the moving two-point discrimination with blunt-pointed tip (MTPDB), and the static two-point discrimination with sharp-pointed tip (STPDS) tests were performed in the order mentioned here. There was a rest period of about 1 minute between the tests using three modalities. For MTPDB testing, the tips of the device were moved in a proximal-distal direction with a length of 3 mm. In the case of the index fingertip, only the STPDB and STPDS tests were performed.

### Two-point discrimination (TPD) sensory testing procedures

**Figure 1:**
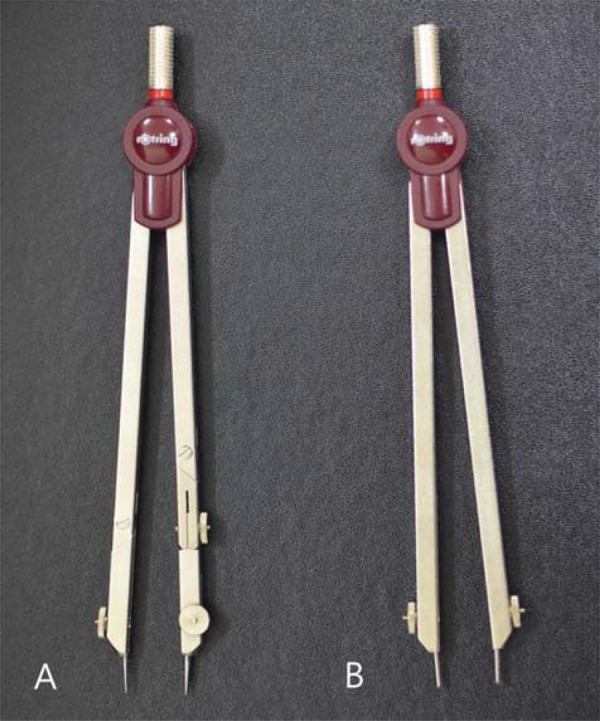
Handheld devices used for measuring two-point discrimination values

The two-point test was performed using a simple hand-operated device, i.e., by drawing compass with blunt or sharp-pointed tip (Figure 1). The interval between the two metal tips of this simple instrument was continuously adjustable and was measured in mm. The two-point test was performed by applying the two tips of the device to the test site.

A; drawing compass with sharp-pointed tip

B; drawing compass with blunt-pointed tip

The stimulus intensity was chosen to be that which the subject could perceive as constant touching or moving without the perception of discomfort or pain for the STPDB and MTPDB tests; i.e., skin blanching itself was not used as control. The STPDS test was performed using a similar procedure to the STPDB and MTPDB tests, but the subjects could perceive mild discomfort due to the sharpness of the tip applied.

The contact time was approximately 1.5 s. The two points of the tool were applied at the same time and perpendicularly to the test surface. The inter-stimulus interval was approximately 5 s for the STPDB and MTPDB tests and 7 s for the STPDS test.

The first distance of the tips, which was large enough for the subject to clearly perceive correctly, was determined at the preliminary testing. The initial TPD test distance was 20 mm for the forehead and cheek, 10 mm for the mentum, 6 mm for both lips and 5 mm for the tongue tip and the index finger tip. If the subject could not correctly perceive the initial distance, a longer distance was set for the initial distance. A threshold was determined using a descending stimulus magnitude and one point was inserted intermittently during the descending series to avoid the subject’s expectation of the continuous decrease in distance between the two points. If the subject answered correctly in response to these changes, the distance decreased in intervals of 1 mm. This testing pattern was continued until the subject answered incorrectly, and the experimenter returned to the next longer distance. The series was terminated when a correct answer for the next longer distance was followed by two incorrect answers on two subsequently shorter distances. This final correct answer was chosen as the end-point for the TPD test. When the subjects continuously had inconsistent responses with the repeated measure of the TPD tests at the given test site, the subjects were excluded from the corresponding test.

Two series of TPD testing for three modalities were performed to determine each TPD value and the mean values of two consecutive measurements were calculated. The subjects were given three alternatives for the answers; i.e., the subject was asked to say “one” if the subject felt one point and “two” if two separate points were felt. If the subject said “I can’t discriminate one or two”, it was regarded as an incorrect answer.

The above tests were carried out in a quiet room at room temperature by one investigator. The orofacial sensitivity tests were conducted with the subjects in the supine position in the dental chair. The subjects were asked to keep their eyes closed throughout the test procedure.

### Data analysis

We defined the test site, sex, and test modality as predictors and the two-point discrimination values as the outcome. Before the data analysis, the normality of the data was evaluated using the Kolmogorov-Smirnov test and data were not normally distributed. Thus, log-transformation of data was applied to perform the further statistic calculation and correct the possible heteroscedasticity. All variables were continuous, and the mean threshold values and standard deviations were calculated from the raw data. The side differences at each test site were analyzed by paired t-test.

To test for the effects of site, sex, and test modality on two-point discrimination, data were analyzed using three-way analysis of variance (ANOVA). When the differences were significant, Tukey *post hoc* analysis was calculated for multiple comparison. The upper limits of normality for a given sample were calculated using the 95% prediction interval (1.96 SD). A 95% upper limit of the confidence interval for the population mean of the TPD test values was calculated according to UCL=μ+SEMxt_0.05_, in which UCL is the upper confidence limit, μ is the sample mean, SEM is the standard error of the mean, and t_0.05_ corresponds to the percentage point of the t-distribution with (n-1) degrees of freedom which results in a two-tailed probability of 0.05. Statistical tests were performed at the 5% significance level. All statistical calculations were performed using the Statistical Package for the Social Sciences (PASW Statistics for Windows, version 18.0, SPSS Inc., Chicago, IL, USA).

## Results

### Subjects

Healthy young adults consisting of 20 men and 20 women were tested. There were no significant age differences between male and female subjects (Independent t test, P=0.327). In the STPDB test, inconsistent responses were recorded for the forehead and cheek for two men and one woman and the tests were excluded (Table 1). Tests performed in four men and one woman on the forehead, two men and one woman on the cheek, and one woman on the mentum were also excluded for the MTPDB test due to inconsistent responses (Table 1). The results of the STPDS tests performed on four men and one woman on the forehead, two men and one woman on the cheek, and one woman on the mentum were excluded due to their inconsistent responses (Table 1).

**Table 1:** Normal values of two-point discrimination test

Test modality			Forehead	Cheek	Upper lip	Lower lip	Mentum	Tongue tip	Index finger
STPDB	M	Mean	16	12.4	4	3.8	6.3	2.8	2.3
		SD	3.2	2.9	1	1	1.4	0.4	0.4
		UL	22.3	18.1	6	5.7	9.1	3.6	3.1
		UCI	17.6	13.8	4.5	4.3	7	3	2.5
		N	18	18	20	20	20	20	20
	W	Mean	14.1	11.3	3.7	3.6	5.7	2.6	2.5
		SD	2.1	1.4	0.6	0.6	1.5	0.5	0.5
		UL	18.2	14.1	4.8	4.7	8.6	3.6	3.4
		UCI	15.1	11.9	4	3.9	6.4	2.8	2.7
		N	19	19	20	20	20	20	20
MTPDB	M	Mean	14.4	10.8	3.4	3.2	5.4	2.4	
		SD	4.1	3	0.8	0.9	1.4	0.5	
		UL	22.4	16.7	4.9	4.9	8	3.4	
		UCI	16.5	12.3	3.8	3.6	6	2.7	
		N	16	18	20	20	20	20	
	W	Mean	12.4	10	3.2	3.2	4.9	2.4	
		SD	2.5	2.1	0.5	0.6	1.6	0.4	
		UL	17.3	14.1	4.3	4.4	8	3.3	
		UCI	13.6	11	3.5	3.5	5.7	2.6	
		N	19	19	20	20	19	20	
STPDS	M	Mean	13.3	10.4	2.9	3	5	2	2.1
		SD	4.3	3.2	1	1.1	1.6	0.4	0.6
		UL	21.7	16.7	5	5.1	7.6	2.9	3.2
		UCI	15.5	12	3.4	3.5	5.8	2.2	2.3
		N	16	18	20	20	20	20	20
	W	Mean	10.8	9.3	3	2.8	4.2	1.9	1.9
		SD	2.2	2.4	0.8	0.9	1.2	0.5	0.6
		UL	15	14	4.5	4.5	6.6	2.8	3
		UCI	11.8	10.5	3.4	3.2	4.8	2.1	2.2
		N	19	19	20	20	19	20	20

STPDB=static two-point discrimination with blunt-tip

MTPDB=moving two-point discrimination with blunt-tip

STPDS=static two-point discrimination with sharp-tip

Abbreviations: SD=Standard Deviation; UL=Upper Limit; UCI=Upper Confidence Interval

Unit of normal value=mm

### Normal values and influence of site, sex, and test modality on two-point perception in the orofacial region

The analyses showed no statistically significant effects of the side on the TPD test values; the means of the right and left side at each site were used for the TPD test threshold values. Descriptive statistics such as mean values and standard deviations were calculated for the test sites and sex in the three different test modalities. Using these data, the 95% upper limits of normal two-point discrimination thresholds in a given sample and the upper confidence limit were also calculated (Table 1). The means and standard deviations of the TPD values in the forehead and cheek were higher than those of the mentum, lips and tongue regardless of sex and test modality.

Three-way ANOVA showed that there were significant differences in the two-point discrimination thresholds depending on the test site, sex, and test modality (Table 2). Significant differences in the two-point discrimination values were observed between the test sites with the descending order from the forehead and cheek>mentum>upper lip and lower lip>tongue tip and index finger (Tukey *post hoc* analysis, P<0.05). Sex differences were also significant and women showed lower two-point discrimination values than those of men (Three-way ANOVA, P=0.001). Test modality showed a significant main effect on the values of the TPD (Three-way ANOVA, P<0.001). The STPDS test measurements were consistently lower than the STPDB and MTPDB test measurements (Tukey *post hoc* analysis, P<0.05). Interactions between two factors or three factors had no significant effect on the TPD thresholds.

**Table 2:** Results of site×sex×modality three-way ANOVA for two-point discrimination

Source	Sum of squares	df	Mean square	F-ratio	P-value	ηp^2^
Site	328.95	6	54.83	593.6	P<0.001	0.877
Sex	0.73	1	0.73	11.72	P=0.001	0.016
Modality	9.52	2	4.76	76.01	P<0.001	0.171
Site˟Sex	0.44	6	0.07	1.17	P=0.319	0.009
Site˟Modality	0.46	11	0.04	0.66	P=0.774	0.01
Sex˟Modality	0.06	2	0.03	0.49	P=0.61	0.001
Site˟Sex˟Modality	0.25	11	0.02	0.36	P=0.97	0.005

Abbreviations: df=degree of freedom; ηp^2^=partial eta squared

## Discussion

The main findings of this study are as follows.

(1) This study showed that there is superior-inferior gradient for spatial acuity in the orofacial region;

(2) Women were more sensitive than men in the TPD perception;

(3) The static TPD with sharp tip seemed to be the most sensitive modality for TPD test.

Two-point perception tests typically express spatial acuity and reflect the density and receptive field size of the low-threshold mechanoreceptors^9^. It is well known that the spatial discrimination ability for touch varies according to the body location^6,19,26^. Weinstein^27^ (1968) found that the fingertip and face had exquisite tactile sensitivity compared to other body sites. Stevens and Choo^19^ (1996) assessed spatial acuity over 13 body regions and showed the superior acuity of the fingertip, lip and tongue. Consistent with these findings, our study exhibited site differences for tactile sensitivity, i.e., the tongue tip was the most sensitive for all TPD modalities as predicted and showed the same range of sensitivity as the index fingertip. The hairy skin, such as the forehead, cheek and mentum, was less sensitive than the glabrous skin including the tongue tip, index finger and both lips.

Psychophysical and microneurography techniques in humans and non-human primates have comprehensively identified the causal relation between stimuli and perception, and the sensory afferents corresponding to perception. Slowly adapting type I fibers (SA I) innervate highly sensitive areas of the skin and exhibit high spatial acuity for tactile stimuli, and the innervation density of SA I afferents is relatively higher in glabrous skin than in hairy skin. On the other hand, rapidly acting (RA) type I fibers have higher sensitivity for movement rather than spatial resolution^1^.

It has been reported that the body regions with high sensitivity have a large density of sensory spots and a low two-point threshold^17^. Using microneurography, Trulsson and Essick^22^ (1997) showed that the major population of low-threshold mechanoreceptors in the tongue are RA and SA units. Similarly, Vallbo and Johansson^23^ (1984) found high density of RA and SA I units in the fingertip. High unitary densities with outstanding spatial resolution in glabrous skin such as tongue tip and fingertip imply that the combined roles of SA I and RA I afferents may enhance tactile acuity. In contrast, predominance of SA afferent has been reported in hairy skin on face, lips and oral mucosa^9^. Trulsson and Essick^22^ (1997) interpreted this finding as a functional adaptation of the mechanoreceptive innervation.

In addition to peripheral factors, high sensitivity of the oral region might be attributed to the cortical representation of oral sensation. It has been demonstrated that the representation of oral sensation over the primary somatosensory cortex is more widely distributed than those for the other body area^18^. The relatively large area of cortical representation of oral region suggests great importance for oral function in human^18^.

There are still two unsolved major problems. First, the TPD test with a handheld instrument does not control for applied pressure^13^. It is well known that controlled stimulus magnitude is one of the prerequisites of psychophysical methods for the assessment of somatosensory function^20^. Moberg^14^ (1990) recommended very light force, 10 to 15 g, which corresponds to the force producing first “blanching” in the skin. Whereas Dellon, Mackinnon and Crosby^7^ (1987) used another method: “just sufficient pressure is utilized for the subject to assess the stimulus”. In fact, Bell-Krotoski and Buford^2^ (1997) indicated that application of force with handheld instrument produces variations and needs to be controlled for test reliability. This lack of repeatability of the force applied may inhibit the reliability of the TPD test. On the other hand, previous studies showed that spatial discrimination in the skin is relatively insensitive to the force applied^16,24,25^. Vriens and van der Glas^25^ (2002) reported that the force levels observed were always at an extremely suprathreshold stimulus intensity and, therefore, the thresholds of TPD were almost invariant in relation to the difference in the force applied. Our study used Dellon’s description for the force applied because there was some difficulty in identifying the very first blanching of the skin. In fact, one of the hardest things that the author experienced during the experiment was applying the two tips of the device with even force or synchronously on the skin surface. The subjects could succeed in the TPD test by recognizing uneven contact, i.e., by recognizing the heavier and lighter application force rather than discriminating between two discrete points. Additionally, false TPD occurred if the two tips of the device were applied to the skin at different times. Thus, we should be careful as to the balance as well as the amount of force applied for reliable measurements despite the inherent uncontrolled force in any handheld tests.

The lack of a standardized protocol to perform TPD tests is another major problem^13^. For example, should the test start with the smallest distance using an increasing method or the widest distance using a decreasing method from the initial distance, and how many correct answers should be used for the value of the TPD? It is widely known that the method of limits leads to systemic errors in estimating thresholds due to response biases, i.e., habituation and expectation^20^. Thus, this study adopted the descending method of limit with intermittent and random insertion of testing stimuli from one to two points as previously reported in Dellon’s study^7^ to reduce the subject biases. Detailed descriptions of the test procedure should become mandatory. In the future, these methodology shortcomings should be clarified.

Sex, as well as the site, influenced the outcome of the TPD tests in the current study. Our findings are in line with previous studies of the face as well as of the hand^3,12,26^. Peters, Hackeman and Goldreich^16^ (2009) hypothesized that this sex difference in somatosensory perception might result from physical differences between men and women. The study showed that tactile perception improves with decreasing finger size and women, on average, have smaller fingers than men. Considering the Merkel cells around the bases of sweat pores^29^, higher density of sweat pores in smaller fingers – which had been proved in Peters’s study – suggests that Merkel receptors are packed more densely in women^16^. Similarly, it is well known that Meissner corpuscles are more densely distributed in smaller fingers^15^. Considering previous studies in the finger, high tactile sensitivity of women in this study suggests increased mechanoreceptor density in orofacial region of women compared to men. However, there are also other studies with contrasting results^4,17^. Considering that site differences were significant for the TPD values in many previous reports despite the different devices, subjects and protocols of those studies, these inconsistent results for the influence of sex on TPD values might imply that sex is not as powerful as site as predictor of TPD values.

Two-point perception was evaluated using three different testing modalities, and static TPD with sharp tip was found to be the most sensitive modality. The difference between the TPD modalities with blunt and sharp tip is the pressure applied. Stimuli with a sharp tip will increase the pressure and might activate the nociceptors^26^. Considering that the significant differences between the STPDS test and the two other modalities were higher in the forehead and cheek than in the lips and tongue tip, the STPDS test rather than the others could be a better modality in the V1 and V2. In particular, this exquisite discriminative ability of the STPDS test would be beneficial in the early detection of sensory recovery in patients with nerve injuries because nociception is commonly regained earlier than touch perception in the course of sensory recovery.

While static two-point discrimination with blunt tip called Weber test is a classic TPD test that evaluates the slowly adapting fiber/receptor system that detects constant touch, the moving two-point discrimination test evaluates the function of the rapidly adapting afferents as a detector of transient touch, i.e., movement^4^. Dellon^4^ (1978) reported that the sensation of moving touch is recovered not only sooner but also to a higher degree than that of constant touch. This suggests that the MTPDB test might be useful in evaluating the extent of sensory recovery, like the STPDS test, in clinical settings.

In the present study, an interesting finding was that most of the subjects often showed inconsistent responses even under the same stimulus condition. In particular, inconsistent responses were prominent in hairy skin on the forehead, cheek and mentum rather than in glabrous skin such as on the lips and tongue. Although it is unclear what actually caused this inconsistency, two factors could be possible explanations. First, the uncontrolled force applied may induce inconsistent responses. However, if we consider that these inconsistent responses were prominent in hairy skin, especially in the forehead and cheek, uncontrolled force alone is not enough for full explanation of these variable responses in hairy facial skin. Second, the neuropsychological aspect of the TPD should be considered. The TPD threshold is influenced by the central nervous system (CNS) as well as by several factors in the peripheral nervous system (PNS)^21^. Tamura, et al.^21^ (2003) indicated that the TPD process is related to evaluation of the distance between the stimuli relative to that of the preceding two-point stimulus, as the conditioning stimulus, as well as the assessment of absolute distance between the stimuli. Thus, the results of our study suggest that the balance between PNS and CNS processing for evaluation of the TPD might be different between hairy and glabrous skin in the orofacial region. In a different point of view, PNS factors might be more influential on the TPD in glabrous skin rather than in hairy skin. Thus, the TPD values should be interpreted with caution in the forehead, cheek and mentum in comparison with the oral region.

Risk of selection bias should be considered as a study limitation. The participants of this study were not representative of the population because they were young students from a dental school. Thus, our normative TPD values cannot be applicable to all ages and the results of this study should be interpreted as a preliminary study.

To our best knowledge, this study was the first to perform TPD test using a drawing compass with blunt or sharp-pointed tip as a simple hand-operated device. The device used in this study, compared to the Disk-Criminator and Aesthesiometer, is simple and affordable enough for use in a clinical setting. In addition, we tested all three trigeminal branches with various TPD modalities.

## Conclusions

The normal TPD values presented in this study suggest heterogeneity of spatial acuity and sex difference in the orofacial region. The cheek and forehead have lower sensory accuracy than other regions evaluated and women were more sensitive than men in TPD perception. Static TPD with sharp tip would be beneficial for the assessment of sensory recovery as well as abnormal sensation in patients with somatosensory abnormalities. Although the TPD test is not recommended as the only tool for evaluation of sensory impairment or sensory recovery, the TPD test using a simple handheld device would provide much more trigeminal sensory information if clinicians use various TPD test modalities with the understanding of the normative values.
